# Predictors of perinatal mortality in emerging regions of Ethiopia: Evidence from EDHS 2016

**DOI:** 10.1371/journal.pone.0322492

**Published:** 2025-05-20

**Authors:** Fikreab Desta, Girma Beressa, Biniyam Sahiledengle, Telila Mesfin, Lemlem Daniel Baffa, Yordanos Sintayehu, Demisu Zenbaba, Daniel Atlaw, Lillian Mwanri

**Affiliations:** 1 Department Public Health, Madda Walabu University, Goba Referral Hospital, Goba, Ethiopia; 2 Research Center for Public Health, Equity and Human Flourishing, Torrens University Australia, Adelaide Campus, Adelaide, South Australia, Australia; 3 School of Medicine, Madda Walabu University, Goba Referral Hospital, Goba, Ethiopia; 4 Department of human Nutrition, University of Gondar, Gondar, Ethiopia; 5 Torrens University Australia, Adelaide Campus, Adelaide, South Australia, Australia; Bahir Dar University College of Medical and Health Sciences, ETHIOPIA

## Abstract

**Background:**

Perinatal mortality rate is one of indictors used to measure the quality of obstetric and pediatric services globally. Compared to developed settings, perinatal mortality rate is higher in low-income countries, indicating societal inequities in health care and a scarcity of prenatal services. It is responsible for roughly 42% of all stillbirth in Sub-Saharan Africa, and 41% of newborn death globally. Despite Ethiopia’s efforts to reduce perinatal mortality by improving the quality of care for maternal and child health, perinatal mortality rate is still very high, and as to our search of pieces of literature there is no study in Emerging regions of the country. Therefore, this study aimed to assess the factors that contribute to perinatal mortality rate in emerging region (Afar, Gambela, Somali, and Benishangul Gumuz) of Ethiopia’s.

**Methods:**

This study relied on data from the 2016 Ethiopian Demographic and Health Survey (EDHS). The analysis included the outcomes of 4, 070 pregnancies with a gestational age of 7 months or more. A multi-level mixed logistic regression analysis was used to examine individual and community-level predictors, accounting for the data’s hierarchical structure. A statistically significant association was determined with a p-value of ≤ 0.05.

**Results:**

Of the 4,070 (weighted) pregnancies in total, 432 (57.36%) children were born to women with a mean age of 28.68 ± 6.53 (ages ± SD). The overall perinatal mortality rate in emerging regions of Ethiopia was 36 deaths per 1,000 pregnancies. The study found that having a birth interval < 2 years (AOR = 3.2, 95% CI: 1.51, 6.59), maternal age greater than or equal to 35 (AOR = 4.3, 95% CI: 1.84, 10.14), drinking an unimproved water source (AOR = 2.7, 95% CI: 1.14, 6.27), and mothers with no education (AOR = 0.33, 95% CI: 0.13, 0.86) were factors significantly associated with a high odds of perinatal mortality rate.

**Conclusion:**

This study revealed a higher perinatal mortality rate as compared to national average. Maternal age, drinking an unimproved water source, and birth interval were significantly associated with perinatal deaths. Despite the enhanced effort to improve maternal and child services, there is still a need for more attention to these interconnected issues helps to reduce effectively the perinatal mortality rate in emerging regions of Ethiopia. Future researchers may benefit from focusing on strong study designs to investigate further the determinants of perinatal mortality, and policymakers good to pay special attention to incorporating the findings into policy.

## Introduction

Perinatal mortality rate is a key indicator that measures the quality of obstetric and pediatric service [1]. Perinatal mortality rates have been reported to be higher in low-income communities compared to their counterparts, indicating societal inequities in health care and a scarcity of perinatal services [2]. The perinatal mortality rate (PMR) was defined as the number of perinatal deaths per 1000 live and stillborn births after 22 weeks of gestation and before seven days after birth [3]. It serves as a crucial indicator of the country’s maternal and neonatal healthcare system [1].

Perinatal mortality is a public health concern; nearly 2 million stillbirths occur globally each year [4]. In 2019, about 2.4 million newborn deaths [4] were reported, with the higher burden in low-income countries [5] when compared to high-income countries, and low-income countries have higher risk of stillbirth and newborn deaths [4,6]. Despite a global decline in stillbirths [4], up to 2019 perinatal mortality rates in Sub-Saharan African (SSA) countries remained high [7,8], accountings for approximately 42% of all stillbirths [4], and 41% of all newborn deaths [6] worldwide. Ethiopia is one of the SSA countries with very high fertility rate (4.6 children per woman) [9].

In 2014, the world launched a platform called “Every Newborn Action Plan (ENAP)” to reduce stillbirths and neonatal deaths to less than 10 per 1,000 total births in countries by 2035 [10]. Ethiopia has been working to reduce perinatal mortality rates by endorsing the “Every Newborn Action Plan”. However, the perinatal mortality rate remains one of Ethiopia’s major public health concerns, with the Ethiopian Demographic and Health Survey (EDHS) reporting 33 perinatal deaths per 1000 births in 2016 [9].

Many studies have identified age of mother [11,12], parity [13–15], attending antenatal care visits [11,16,17], skilled birth attendants [13], low family income [11,18], place of delivery [19,20], birth interval [18,21], residency [15], access to clean water supply [22], access to participation in decision making [18], previous history of perinatal mortality as significant factors determining perinatal mortality rates.

Despite Ethiopia’s efforts to reduce perinatal mortality rates by ensuring health equity and improving the quality of care for maternal and child health [12]. There is still a very high portion of perinatal mortality rate in Ethiopia [23]. A few studies have been conducted in Ethiopia to identify risk factors for perinatal mortality rate [13–15,19,24,25]. Furthermore, a previous study was limited to areas of Ethiopia with small populations, resulting in findings that misrepresented the entire country. National data on the predictors influencing the rates of perinatal mortality in the various regions are scarce. The primary goal of this study was to use nationally representative data to assess individual and community-level predictors of perinatal mortality rates in Ethiopia’s emerging regions, allowing the findings to inform critical policy and practice drivers for reducing the scourge of high perinatal mortality rates in Ethiopia’s emerging (developing) regions.

## Methods

### Data source and sampling procedures

The Ethiopian Demographic and Health Survey (EDHS) data from 2016 were used. The Ethiopian Central Statistical Agency (ECSA) collected data for the EDHS, which had a cross-sectional design, between January 18 and June 27, 2016. Nine geographical regions and two administrative cities make up Ethiopia’s administrative divisions. Kebeles, the smallest administrative subdivision, is further divided into districts. Kebeles are divided into census enumeration areas (EAs), which makes it easier to conduct surveys.

A stratified, two-stage cluster sampling was used. The EDHS-2016 sampling frame was Ethiopia’s 2007 ECSA Population and Housing Census (PHC). The census frame includes all of the 84,915 enumeration areas (EAs) created for the 2007 PHC. Households served as secondary sampling units (SSU), while EAs were the primary sampling units (PSU). The 2016 EDHS contains 645 EAs, with 202 in urban areas and 443 in rural areas. The second stage of selection involved choosing a fixed number of 28 households per cluster, with an equal chance of systematic selection from the newly created household list. The study included 4070 mothers from emerging regions of Ethiopia (Afar, Somali, Benishangul and Gambela). As a result, the outcomes of 4070 pregnancies that reached 7 months of gestational age (including twin pregnancies) were examined in order to identify factors influencing perinatal mortality rate. The majority of pregnancy and postnatal care variables in EDHS data were only collected for the most recent birth or pregnancy.

### Variables of the study

#### Outcome variable.

The perinatal mortality rate, an outcome variable, was calculated by dividing all births (including stillbirths) with a pregnancy duration of seven months or more during the five years prior to the survey. This rate included both stillbirths and early neonatal deaths. When a pregnancy is ended after seven months or more, a stillbirth occurs. Early neonatal death (days 0–6) is defined as death within seven days. The outcome variables were coded as “0” in cases where perinatal mortality did not occur and “1” in those cases.

#### Independent variable.

Individual and community level factors were the independent variables for this study. The independent variables were chosen based on a review of the literature [26–28]. Maternal age (in years), maternal education status, Sex of household head, wealth index, age at birth, place of delivery, birth interval, time to breastfeeding Initiations, previous history of a terminated pregnancy, tetanus toxoid vaccination, water facility, and health insurance were considered as individual factors. Factors such as residence, distance from health facilities, and times to reach the water source were considered as community-level factors.

The women’s health care decision-making autonomy was assessed as the person who usually decides to obtain healthcare. Which was categorized as women participating in making health care decisions and didn’t participate in making health care decisions (decided by their husband/ partner). The water source was leveled as improved if the source of drinking water comprises one of the following: a piped household connection, public standpipe/borehole, protected dug well or spring, and/or rainwater collection. The toilet facility was leveled as improved if it has (piped into dwelling, piped to yard/plot, piped to a neighbor, and public tap/standpipe) and not improved for (no facility/bush/feld, composting toilet). wealth index, in the original it was categorized into five wealth quintiles: ‘poorest’, ‘poor’, ‘middle’, ‘rich’, and ‘richest’. For this study, we re-coded the wealth index into two categories as ‘poor’ (poor, very poor and middle), and ‘rich’ (rich and very rich) to obtain an adequate sample in each category.

### Data management and analysis

Before conducting any statistical analysis, the data were checked for completeness and weighted. STATA Version 14 was used for the data analysis. Because of the sample’s non-proportional allocation to different regions, urban and rural areas, and potential differences in response rates, proportions and frequencies were estimated after applying sample weights to the data to account for disproportionate sampling and nonresponses, as recommended by the EDHS. The EDHS report contains a detailed explanation of the weighting procedure [9].

Due to the sampling techniques used in the EDHS (multi-stage stratified cluster) data, a two-level data hierarchy was considered in this study. Individual pregnant mothers in households were level one units, and enumeration areas (clusters) were level two units. The first level of enumeration areas (pregnant mothers in households) was nested at the next higher level of enumeration areas (community).

We utilized a complex sample survey multilevel data analysis technique (melogit [pweight=swt] || v001:) to take a thorough approach to analyzing complex survey data. Using this methodology, we addressed the complexities of survey design while also ensuring the provision of reliable statistical inferences. A multilevel mixed logistic regression model was then used to identify the individual and community-level factors that contribute to perinatal mortality. Bivariable and multivariable multilevel logistic regression analyses were carried out to examine the association between the predictors and outcome variable. Variables with a P-value of less than 0.25 were considered for a multivariable multilevel logistic regression analysis. Statistical significance was declared at a P-value < 0.05.

The random effects (variation of effects) were evaluated by intra-cluster correlation (ICC) and proportional change in variance (PCV), which measure the variability between clusters in the multilevel logistic regression model [29–31]. The ICC explained the cluster variability. In the multilevel logistic regression model, PCV can measure the total variation due to factors at the community and individual levels factors [30]. Median odds ratio (MOR) can quantify unexplained cluster variability (heterogeneity). MOR converts cluster variance into OR scale in the multilevel model [29,30]. $MOR=exp(\sqrt{(2}\times V)\times 0.6745)\approx exp(0.95\sqrt{V}$ Where V is the estimated variance of clusters and PCV were determined using the estimated variance of clusters using the following formula $ICC=\frac{V}{V+\;\frac{\pi^{2}}{3}\;}$ Where V denotes community variance, and π^2^/3 denotes individual-level variance that is fixed for log distribution (equal to 3.29) and $PCV=\frac{(Va-Vb)\times 100}{Va}$ where Va = variance of the initial (null) model; Vb = variance of the model with more terms. To identify the potential predicators with perinatal Mortality; a multilevel mixed effect binary logistic regression was fitted. Four models have been developed with the assumption of varying intercepts across communities (clusters) but fixed coefficients. The frst was the null model (Model I) fitted without predictors variables. The second model (model II) was fitted for individual-level factors and conducted to examine the contribution to the variation of perinatal mortality. Whereas the third model (Model III) was adjusted for community-level factors and used to examine the contribution of variation in perinatal mortality across the cluster. Finally, the fourth model (Model IV) was developed by combining individual and community-level factors to associated with perinatal mortality rates. Model comparison was made using Akaike information criteria (AIC) and Bayesian information criteria (BIC). The Variance Inflation Factors (VIF) and tolerance were used to test for multicollinearity and its value is less than 10 and greater than 0.1 respectively.

### Ethical consideration

The Ethiopian Demographic and Health Survey was carried out with the approval of the Ethiopian National Research Ethics Review Committee. Permission to use the 2016 EDHS database for further analysis was obtained from http://www.dhsprogram.com. Every strategy adhered to the pertinent Helsinki Declaration principles.

## Results

### Socio-demographic and economic characteristics

Among the 4070 (weighted) pregnancies, 230.54 (30.64%) of the mothers were the heads of their households; approximately 3,489 (85.72%) of the mothers were from rural areas; and 2,986 (73.37%) of the mothers had never attended college. In Ethiopia’s developing regions, the overall perinatal mortality rate per 1000 births was 32. Mothers who were over 35 years old (34.94 per 1000), had the lowest wealth index (73.99 per 1000), and were not exposed to the media (50.79 per 1000) had the highest rate of perinatal mortality (Table 1).

**Table 1:** The perinatal mortality among pregnant women by socio-demographic and economic characteristics in emerging regions, Ethiopia, 2016.

Variable	Frequency (%)	Weighted frequency (%)
**Maternal age (years)**		
Less than 20	186 (4.57)	27.98 (3.72)
20-24	923 (22.68)	160.3 1(21.36)
25-29	1205 (29.61)	240.43 (31.95)
30-34	852 (20.93)	148.97 (19.80)
>=35	904 (22.21)	174.43(23.18)
**Residance**		
Urban	581 (14.28)	108.66 (14.44)
Rural	3489 (85.72)	643.56 (85.56)
**Maternal educational status**		
No education	2,986 (73.37)	602.51 (80.07)
Primary education	768 (18.87)	115.78 (15.39)
Secandary education	316 (7.76)	34.22 (4.55)
**Sex of household head**		
Male	2819 (69.26)	521.91 (69.36)
Female	1251 (30.74)	230.54 (30.64)
**Wealth index**		
poorest	2619 (64.35)	490.97 (65.24)
poorer	433 (10.64)	75.41 (10.02)
middle	296 (7.27)	59.49 (7.91)
Rich	285 (7.00)	45.65 (6.07)
Richest	437 (10.74)	80.97 (10.76)
**Imroved water source**		
Unimproved water source	1886 (46.34)	395.39 (52.54)
improved water source	2184 (53.66)	357.12 (47.46)
**Improved toilet facility**		
Unimproved water source	3533 (86.81)	602.32 (80.12)
Improved water source	537 (13.19)	150.19 (19.96)
**Time to reach water source**		
water on premises	430 (10.57)	96.30 (12.80)
>=30 minutes	1933 (47.49)	330.18 (43.88)
<30 minutes	1707 (41.94)	326.04 (43.33)

Variables measured at the time of the survey: residence, education status, wealth index, sex of household, water source, and toilet facility. Variables measured at the time of birth: maternal age (years)

PMR: perinatal mortality rate.

### Reproductive characteristics and perinatal mortality rates

Of the 4,070 (weighted) pregnancies in total, 432 (57.36%) were born to women who were between the ages of 20 and 29. Mothers who gave birth at home (591.60; 78.84%), mothers with a birth interval of less than two years (66.15 per 1000), mothers who have a significant issue with distance from the medical facility (56.93 per 1000), and all pregnant mothers without health insurance had higher rates of perinatal mortality (Table 2).

**Table 2:** The perinatal mortality among pregnant women by reproductive health characteristics of study participants in emerging regions, Ethiopia, 2016.

Variable	Frequency (%)	Weighted frequency
**Mothers age at birth**		
< 20	643 (15.80)	105.70 (14.05)
20-29	2289 (56.24)	431.67 (57.36)
30-39	1025 (25.18)	191.16 (25.40)
40-49	113 (2.78)	23.98 (3.19)
**Place of delivery**		
home	3160 (77.79)	591.60 (78.84)
Health institution	902 (22.21)	158.80 (21.16)
**Preceding birth interval**		
< 2 years	1224 (37.00)	272.37 (43.40)
≥ 2 years	2084 (63.00)	355.17 (56.60)
**Time to breast feeding initiation**		
within 1 hour	2599 (66.98)	533.68 (74.30)
< 1 hour	1281 (33.02)	184.64 (25.70)
**Distance from health facility**		
big problem	2398 (58.92)	414.62 (55.10)
not big problem	1672 (41.08)	337.90 (44.90)
**Health insurance**		
No	4063 (99.83)	751 (99.90)
Yes	7 (0.17)	0.74 (0.10)
**Every had terminated preginancy**		
No	3735 (91.77)	688.60 (91.50)
Yes	335 (8.23)	63.97 (8.50)
**Smokes cigarette**		
No	4014(98.62)	746.92 (99.26)
Yes	56(1.38)	5.59 (0.74)
**Currently residing with husband/partener**		
living with her	3118(80.82)	588.60 (81.70)
staying elsewhere	740 (19.18)	131.85 (18.30)

Variables measured at the time of birth: age of mother at birth, place of delivery, birth interval,cigarette smoking, time to breastfeeding initiations, and history of terminated pregnancy. Variables measured at the time of the survey: health insurance, distance from health facility

PMR: perinatal mortality rate

### The prevalence of perinatal mortality rate among mothers of emerging regions in Ethiopia, EDHS, 2016

**Fig 1:**
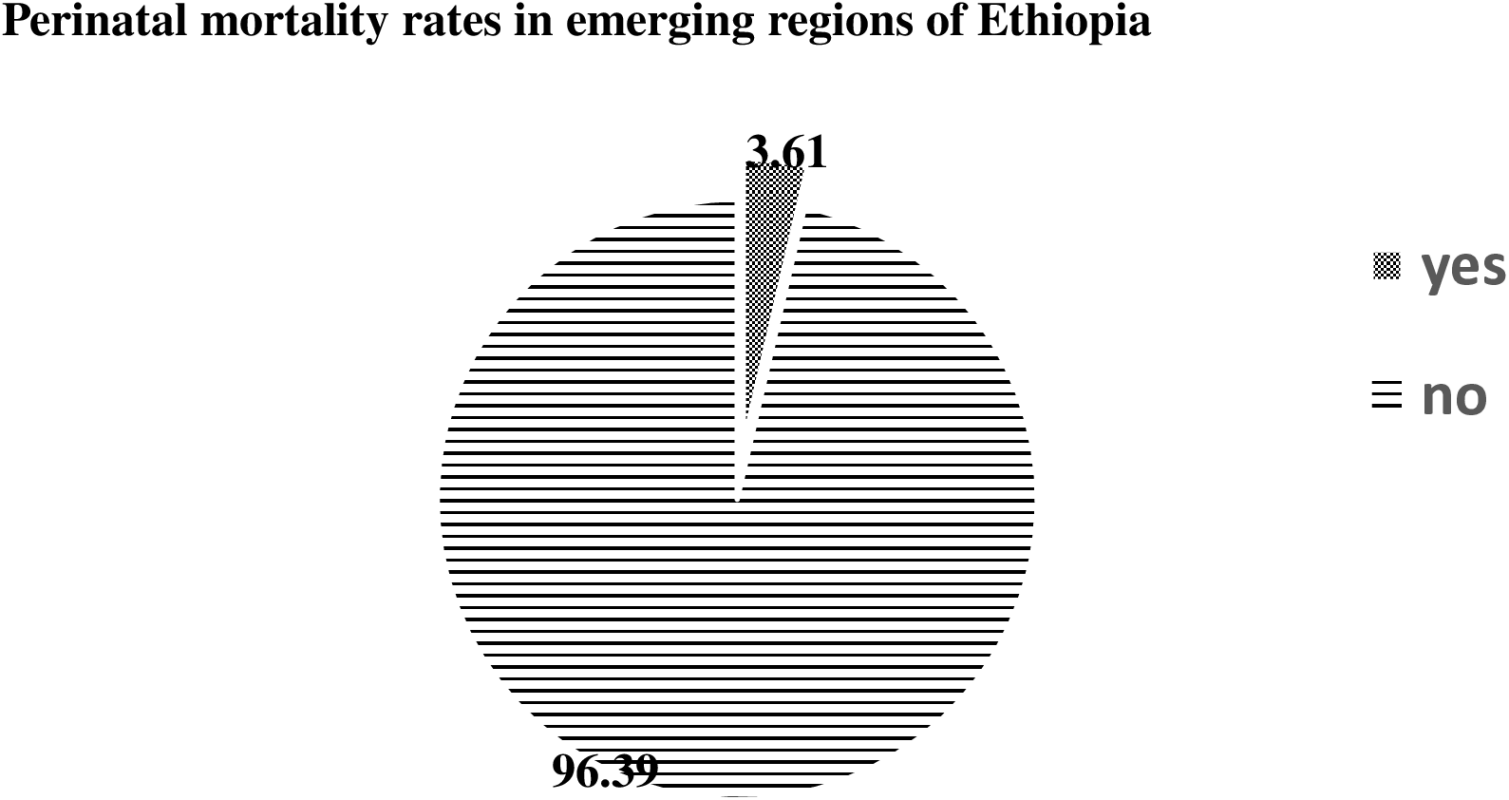
Perinatal mortality rates among pregnancies in emerging regions of Ethiopia, EDHS 2016.

Of the 4,070 pregnancies in emerging regions of Ethiopia, the perinatal mortality rate was 36 deaths per 1,000 pregnancies (Fig 1).

### Comparison and identification of the appropriate model for potential factors associated with perinatal mortality

A complex multivariable multilevel mixed effect binary logistic regression was fitted to identify the potential factors associated with perinatal mortality. The final model (Model IV) was appropriate to identify the individual and community level factors of perinatal mortality in emerging regions in Ethiopia after checking the model fitness level using different post estimation methods (AIC, BIC and loglikelihood). The largest values of Log-likelihood were observed in Model VI, and this implies that Model VI for perinatal mortality was a better explanatory model.

## Factors associated with perinatal mortality rates

A multilevel mixed effect binary logistic regression was fitted to identify the potential factors associated with perinatal mortality. The final model (Model IV) was appropriate to identify the individual and community level factors of perinatal mortality in emerging regions in Ethiopia after checking the model fitness level using different post estimation methods (AIC, BIC and loglikelihood) (Table 3).

**Table 3:** Variability at community-level and model comparison for perinatal mortality among women’s in the 5 years preceding the survey in Ethiopia, EDHS, 2016.

Parameters	Null model	Model II	Model III	Model IV
ICC	9.58%	2.65E-33	7.40%	1.86E-32
AIC	264.93	120.05	269.23	112.98
BIC	271.24	217.64	300.78	187.61
Log-likelyhood	-131.46	-43.49	-129.62	-43.03
PCV	Reference	99.50%	52.10%	99.77%
MOR	1.6	1.4	1.45	1.37

ICC: intra-cluster correlation; PCV: Proportional Change in Variance; AIC: Akaike’s Information Criterion; BIC: Bayesian Information Criteria

Null model: a model with no independent variables

Model II: a model with only individual/household-level factors

Model III: a model with only community-level factors

Model IV: a model with both individual and community-level factors

The multivariable multilevel logistic regression analysis revealed that birth interval, the age of women, the water source, and educational status of women in the community were statistically significant factors of perinatal mortality of emerging regions in Ethiopia (Table 4). The odds of perinatal mortality among mothers having birth interval less than two years were 3.2 times higher than having two and above years birth interval [AOR: 3.2; 95% CI: 1.51, 6.59; at *P-*value=0.002]. The odds of perinatal mortality among mothers of age less than 25 years were also 3.9 times higher than mothers having age of greater than 35 years [AOR: 3.9; 95% CI: 1.71, 9.12 at *P-*value = 0.001]. The odds of perinatal mortality were 2.7 times higher [AOR: 2.7, 95% CI: 1.14, 6.72 at *P-*value=0.024] among mothers who are using unimproved water source as compared to their counterpart.

**Table 4:** The multivariable multilevel logistic regression analysis of factors associated with perinatal mortality in emerging regions, Ethiopia, 2016.

Variables	Null	Model II AOR at 95% CI	Model III AOR at 95% CI	Model IV AOR at 95% CI
**Maternal age (years)**				
less than 35		1 (Ref)		1 (Ref)
≥ 35		1.8 (1.11, 3.03)^*^		4.3 (1.84, 10.14)^**^
**Sex of household head**				
Male		0.75 (0.19, 2.89)		0.79 (0.21, 2.98)
Female		1 (Ref)		1 (Ref)
**Maternal educational status**				
No education		1.01 (0.62, 1.66)		0.33 (0.13, 0.86)^**^
primary and above		1 (Ref)		1(Ref)
**Wealth index**				
poor		1.9 (0.47, 7.67)		1 (0.28, 3.66)
rich		1 (Ref)		1 (Ref)
**Birth interval**				
< 2 years		3.2 (1.53, 6.65)^*^		3.2 (1.51, 6.59)^**^
≥ 2 years		1 (Ref)		1 (Ref)
**Delivery palce**				
home		2 (0.55, 7.36)		1.93 (0.55, 6.83)
health institution		1 (Ref)		1 (Ref)
**Breast feeding intiatio time**				
within one hour		1 (Ref)		1 (Ref)
greater than one hour		0.52(0.16, 1.68)		1.2 (0.42, 3.46)
**Ever had terminated pregnancy**				
No		1.27 (0.48, 3.35)		0.79 (0.21, 2.98)
Yes		1 (Reference)		1(Reference)
**Decision making autonomy**				
Yes		0.73(0.32, 1.68)		0.71 (0.31, 1.64)
No		1 (Ref)		1(Ref)
**Livining with husaband**				
Yes		0.95 (0.13, 6.86)		0.91 (0.13, 6.57)
No		1 (Ref)		1(Ref)
**Water source**				
unimproved		3.1 (1.3, 7.22)^**^		2.67 (1.14, 6.27)^**^
improved		1 (Ref)		1 (Ref)
**Toilet type**				
unimproved		0.43 (0.19, 0.97)^**^		0.43 (0.18, 1.06)
improved				1(Ref)
**Type of residency**				
Urban			1 (Ref)	1 (Ref)
Rural			3.5(1.52, 8.07)^**^	5 (0.94, 26.98)
**Time to reach water source**				
On premises			1 (Ref)	1 (Ref)
≤ 30minutes			0.51 (0.24, 1.11)	0.67 (0.14, 3.21)
> 30 minutes			0.74 (0.36, 1.53)	0.92 (0.25, 3.49)
**Time to reach health facility**				
big problem			0.96 (0.62, 1.49)	0.98 (0.43,2.19)
not big problem			1 (Ref)	1 (Ref)

*Statistically significant p value < 0.05;

**P-value < 0.001; Ref: Reference; AOR: adjusted odds ratio; CI: confidence interval; SE: standard error; ICC: intra-class correlation, PCV: proportional change in variance; AIC: Akaike’s information criterion; BIC: Bayesian information criteria.

## Discussion

A multilevel mixed effect binary logistic regression was used to identify potential predictors for perinatal mortality in emerging regions in Ethiopia. The findings revealed that the mother’s age, birth interval, and water source were all significant predictors of perinatal mortality and maternal educational status has showed negative association. Ethiopia has one of the world’s highest perinatal mortality rates [21]. Our results revealed 36 perinatal deaths per 1000 livebirths found in emerging regions of Ethiopia. This result was higher than that of national level perinatal mortality (33 deaths per 1,000 livebirths) [32] and a systematic review conducted in sub-Saharan Africa (34.7 deaths per 1000 livebirths) [7]. The possible reason might be these regions are areas characterized by dispersed pastoralists and semi-pastoral communities who live in extreme poverty with little access to health care. As a result, policymakers and non-governmental organizations working in these regions focus on this problem. According to the findings of the current study, mothers above the age of 35 years have a higher risk of perinatal mortality than their counterparts. This result is consistent with the study [33–37]. These may be related to the aging process itself or increased risk or coexisting factors such as multiple gestational and chronic medical conditions [33,38] along with obstetrical complications such as intrauterine growth restriction, placental abruption, preterm labor, and preeclampsia [39,40].

The odds of perinatal mortality were 2.67 times higher in women who used an unimproved water source than their counterparts. These results are consistent with findings studied and conducted in Ethiopia [41,42], Pakistan [43] and Nigeria [44]. We hypothesize that the reasons for high odds of perinatal deaths among women consuming water from unimproved sources could be that unimproved water sources are more likely to be contaminated and less likely to prevent the spread of water-borne diseases such as intestinal parasites, bacterial, and other pathogenic conditions [45]. In this study, mothers who gave birth within two years of each pregnancy had a higher risk of perinatal mortality than those who gave birth after the interval of two years of pregnancies. These findings are in conformity with previously conducted studies [14,21,24,46,47] where shorter birth intervals have been reported to negatively impact women’s nutrition for recovery, endometrial healing, cervical competency, and optimal breast lactation, ultimately affecting subsequent pregnancy outcomes [28,48]. This study found a negative relationship between maternal educational status and perinatal mortality. As far as we know, no one else has reported on this relationship. The possible justification could be due to confounding, and as observed during data cleaning, there was a low response rate in part of formal education, indicating the need for future studies to investigate this further.

### Strength and limitation of the study

The EDHS is a nationally representative household survey data with a high response rate, and the findings are applicable at national level. The survey results are also useful for policymakers and program managers, providing insights into the implementation of effective intervention strategies at the national and regional levels. Furthermore, this study used multilevel modeling to account for the EDHS data set’s hierarchical structure. However, because the data came from a cross-sectional survey, it is difficult to determine causal effect relationships.

## Conclusion

Perinatal mortality rate in emerging regions of Ethiopia is higher than the national average. The findings suggest that the government and other stakeholders should pay further attention on mothers: in advanced ages (greater than or equal to 35 years) and who use unimproved water source. Additionally, special focus should also be paid in women who have shorter spacing between births to reduce perinatal mortality rate. Community-based outreach activities and public health interventions focusing on improving sources of drinking water and providing health education on spacing births should be amplified. Finally, policymakers and governments service should enhance policy and practice drives that address both the community-level factors and individual factors in order to achieve the Sustainable Development Goals (SDG) targets and goals by 2030.

## Supporting information

S1 Data PNM mini data for plose. (xls)
